# Direct binding of calmodulin to the cytosolic C-terminal regions of sweet/umami taste receptors

**DOI:** 10.1093/jb/mvad060

**Published:** 2023-08-01

**Authors:** Atsuki Yoshida, Ayumi Ito, Norihisa Yasui, Atsuko Yamashita

**Affiliations:** Graduate School of Medicine, Dentistry and Pharmaceutical Sciences, Okayama University, Okayama, Japan; Graduate School of Medicine, Dentistry and Pharmaceutical Sciences, Okayama University, Okayama, Japan; Graduate School of Medicine, Dentistry and Pharmaceutical Sciences, Okayama University, Okayama, Japan; Graduate School of Medicine, Dentistry and Pharmaceutical Sciences, Okayama University, Okayama, Japan

**Keywords:** calmodulin, cytosol, sweet taste, taste receptor type 1, umami taste

## Abstract

Sweet and umami taste receptors recognize chemicals such as sugars and amino acids on their extracellular side and transmit signals into the cytosol of the taste cell. In contrast to ligands that act on the extracellular side of these receptors, little is known regarding the molecules that regulate receptor functions within the cytosol. In this study, we analysed the interaction between sweet and umami taste receptors and calmodulin, a representative Ca^2+^-dependent cytosolic regulatory protein. High prediction scores for calmodulin binding were observed on the C-terminal cytosolic side of mouse taste receptor type 1 subunit 3 (T1r3), a subunit that is common to both sweet and umami taste receptors. Pull-down assay and surface plasmon resonance analyses showed different affinities of calmodulin to the C-terminal tails of distinct T1r subtypes. Furthermore, we found that T1r3 and T1r2 showed the highest and considerable binding to calmodulin, whereas T1r1 showed weaker binding affinity. Finally, the binding of calmodulin to T1rs was consistently higher in the presence of Ca^2+^ than in its absence. The results suggested a possibility of the Ca^2+^-dependent feedback regulation process of sweet and umami taste receptor signaling by calmodulin.

## Graphical Abstract

**Graphical Abstract:**
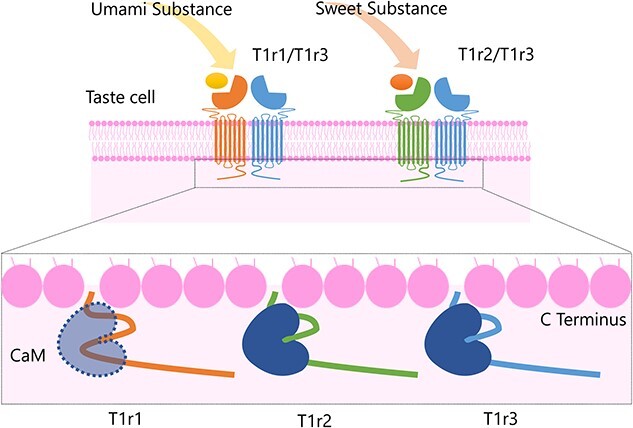

## Abbreviations

BSA: bovine serum albumin
CaM: calmodulin
SPR: surface plasmon resonance
T1r: taste receptor type 1

**Fig. 1:**
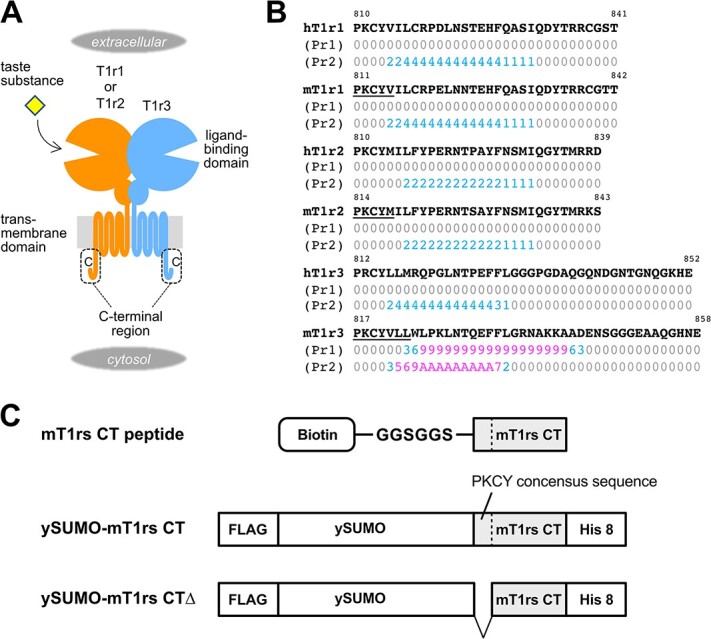
**Schematic of the cytosolic C-terminal regions of T1rs. (A)** Schematic drawing of the overall architecture of T1r. The C-terminal regions present in the cytosol are enclosed with dashed lines. **(B)** Amino acid sequences of the C-terminal regions of T1rs from human (hT1rs) and mouse (mT1rs). The scores of two distinct prediction tools, the Calmodulin Target Database *(**31**)* and the Calmodulation database and Meta-analysis predictor *(**32**)* servers, are provided below the amino acid sequences and are labeled as Pr1 and Pr2, respectively. Sequences containing the consensus motif PKCY that were deleted in FLAG-ySUMO-mT1rs CTΔ constructs are underlined. **(C)** Schematic illustration of the peptides and ySUMO-fusion constructs used in this study.

Taste receptors are responsible for the perception of chemical substances in food and for the transmission of chemical signals into taste sensory cells present in the oral cavity *(**1**,**2**)*. Therefore, these receptors are equipped with sites that can interact not only with extracellular chemicals but can also interact with molecules that participate in intracellular signaling. For example, sweet and umami taste receptors exist in taste cell membranes and possess a specific region for chemical recognition on their extracellular side. The receptors consist of heterodimeric pairs of taste receptor type 1 (T1r) proteins, namely T1r1, T1r2 and T1r3; and T1r1/T1r3 and T1r2/T1r3 heterodimers serve as umami and sweet taste receptors, respectively *(**3–5**)* (Fig. 1A). T1r proteins belong to the class C G protein-coupled receptor (GPCR) family, which is characterized by the presence of a large ligand-binding domain (LBD) on their extracellular side *(**6**,**7**)*. Structural analyses of class C GPCRs have emphasized the importance of agonist binding to the LBD for the signal transmission process. In this process, an agonist binding to the LBD induces a conformational rearrangement of the LBD dimer *(**8**)*. The conformational change further transmits a signal to the downstream heptahelical transmembrane domain to rearrange the heterodimeric configuration, thereby resulting in receptor activation and enabling G-protein coupling *(**6**,**9**,**10**)*. In the case of T1rs, major taste substances for sweet and umami receptors, including sugars, many artificial sweeteners, amino acids and nucleotides, are known to be recognized at LBDs *(**11–13**)* and can therefore induce conformational rearrangement of this region *(**14**)*.

G-protein activation by T1rs is followed by activation of phospholipase *β*C, and the resulting production of inositol 3-phosphate (IP_3_) activates an IP_3_ receptor at the endoplasmic reticulum (ER). This in turn allows Ca^2+^ release from the ER, resulting in an elevation of cytosolic Ca^2+^ concentration and in taste cell depolarization *(**15–18**)*. However, the signal transduction process of T1r on the cytosolic side of the taste cell has not been as extensively studied compared with the processes that occur on the extracellular side. In particular, little is known about the cytosolic molecules that regulate T1r function, with only a few examples having been reported *(**19**,**20**)*.

A representative cytosolic protein that participates in the regulation of receptor proteins is calmodulin (CaM). CaM is a 16.7 kDa protein possessing specific Ca^2+^ binding sites, and is ubiquitous in all eukaryotic cells, where it is related to various Ca^2+^-mediated physiological processes *(**21–23**)*. Ca^2+^ binding to CaM induces conformational changes that permit interaction with target molecules in a Ca^2+^-dependent manner. Given these molecular properties, CaM regulation of the function of many receptors is dependent on cytosolic Ca^2+^ concentration. For example, metabotropic glutamate receptors (mGluRs), a receptor family of class C GPCRs, have CaM binding sites at its cytosolic C-terminal regions *(**24**)*. Moreover, it has also been reported that signaling *(**25–27**)* and membrane trafficking *(**28**,**29**)* of several subtypes of mGluRs are regulated by direct binding of CaM. In terms of taste signaling, CaM expression has been observed in taste buds, the organ where taste receptors exist *(**30**)*. Nevertheless, the relationship between CaM and T1rs has not been well characterized.

In this study, we analyse the interaction between the cytosolic C-terminal regions of sweet and umami taste receptors and CaM. We observed Ca^2+^-dependent binding of CaM to the cytosolic region of mouse T1r3, the subunit that is common to both sweet and umami taste receptors. Our results suggest the possibility of a Ca^2+^-dependent feedback regulation process of taste receptor signaling by CaM.

## Materials and Methods

### Calmodulin-binding site prediction

The amino acid sequences of the C-terminal cytosolic regions in T1rs (Fig. 1B) were used to predict calmodulin binding sites by the Calmodulin Target Database *(**31**)* and the Calmodulation database and Meta-analysis predictor *(**32**)* servers. The boundaries between transmembrane regions and cytosolic regions downstream of the last transmembrane helix VII in human (h) and mouse (m) T1rs (UniProt IDs: hT1r1, Q7RTX1; mT1r1, Q99PG6; hT1r2, Q8TE23; mT1r2, Q925I4; hT1r3, Q7RTX0; mT1r3, Q925D8) were predicted using the TMHMM server *(**33**)*.

### Preparation of mCaM protein

A DNA fragment coding mouse calmodulin (mCaM) was amplified by PCR using an MGC cDNA clone, pXY-Asc-mCaM1 (clone ID: 6838207), as a template. The purified PCR product was then cloned into a pET25b vector using NcoI and NheI sites to produce pET25b-mCaM1.

Next, *Escherichia coli* strain BL21 (DE3) pLysS was transformed with pET25b-mCaM1 then cultured in LB medium (Nacalai Tesque) containing 34 μg/ml chloramphenicol and 100 μg/ml carbenicillin at 37°C until the OD_600_ value reached 0.6–0.8. Protein expression was then induced by adding 0.1 mM isopropyl-β-d-thiogalactopyranoside (IPTG), followed by further culturing at 37°C for 8 h, after which cells were harvested by centrifugation. mCaM was then purified according to the method described by Hayashi *et al*. *(**34**)*. Briefly, cells were suspended in lysis buffer (50 mM Tris–HCl, 2 mM EDTA and 0.2 mM PMSF, pH 7.5) then lysed by sonication on ice. The soluble fraction was collected by centrifugation, added with CaCl_2_ to a final concentration of 5 mM_,_ then applied to a Phenyl Sepharose Fast Flow (high sub) column (GE Healthcare Life Sciences) equilibrated with 50 mM buffer A (Tris–HCl, 0.1 mM CaCl_2_, 0.1 M NaCl, pH 7.5) at room temperature. After washing the column with 5 C.V. of buffer A, followed by 8 C.V. of buffer B (50 mM Tris–HCl, 0.1 mM CaCl_2_, 0.5 M NaCl, pH 7.5), the protein fraction was eluted in buffer C (50 mM Tris–HCl, 1 mM EGTA, pH 7.5).

### Preparation of ySUMO-fusion proteins

To prepare fusion proteins, we first constructed an expression vector, pNySUMO-bc, which encodes FLAG-ySUMO. First, the pHFT-ySUMO vector was used as a template to amplify the DNA fragment coding ySUMO by PCR *(**35**)*. The resulting PCR product was then subcloned into pNGFP-bc*(**36**)*, a pET22-based vector, using NdeI and XhoI sites to produce pNySUMO-bc. Next, we constructed a series of expression vectors (named pNySUMO-bc-T1rs CT) that encoded the ySUMO-T1rs CT and contained an octa-histidine tag at the C-terminus. To this end, the DNA fragments encoding T1rs CT-octa-histidine tag segments were subcloned into pNySUMO-bc using BamHI and XhoI restriction sites. To generate the FLAG-ySUMO fusion of mT1rs CTΔ, in which the N-terminal PKCY consensus sequences of mT1rs CT were truncated (Fig. 1C, *bottom*), inverse PCR was carried out using the pNySUMO-bc-T1rs CT vectors as a template. Next, BL21 (DE3) cells were transformed with these expression vectors then grown at 37°C in LB media containing 100 μg/ml carbenicillin until the OD_600_ value reached 0.5–0.8. Expression was induced by adding 1 mM IPTG and shaking the culture at 20°C overnight. Cells were then pelleted by centrifugation, resuspended in equilibration buffer (20 mM Tris–HCl, pH 8.0, 500 mM NaCl, 10 mM imidazole) supplemented with cOmplete EDTA-free Protease Inhibitor Cocktail (Roche) and subsequently lysed via sonication. Cell lysates from the soluble fraction were used for protein purification by Ni-NTA Superflow (Qiagen). After washing the beads with a wash buffer (20 mM Tris–HCl, pH 8.0, 500 mM NaCl, 50 mM imidazole), proteins were eluted in an elution buffer (20 mM Tris–HCl, pH 8.0, 500 mM NaCl, 500 mM imidazole). Protein concentrations were estimated via Bradford assay using a Coomassie (Bradford) Protein Assay Kit (ThermoFisher, cat no. 23200) with BSA used as a standard.

### Pull-down assays

Chemically synthesized peptides of the cytosolic C-terminal region of T1rs with ~90% purity were obtained from Hokkaido System Science (Sapporo, Japan). These peptides contained an N-terminal GGSGGS linker segment and were biotinylated at the N-terminus (Fig. 1C, *top*). Streptavidin MagneSphere Paramagnetic Particles (40 μg, Promega) were washed twice with HBS-T (20 mM HEPES-Na, 150 mM NaCl, 0.005% Tween 20, pH 7.9) then mixed with 300 μl of 3 μM biotinylated peptide in HBST containing 10 mM TCEP. This mixture was rotated at 4°C for 1 h. The beads were separated using a MagneSphere magnetic separation stand (Promega) then washed with HBS-T twice. Next, the beads were buffer-exchanged with a Ca^2+^-free (−Ca^2+^) buffer (20 mM HEPES-Na, 150 mM NaCl, 0.005% Tween 20, 1 mM EGTA, pH 7.9) or a Ca^2+^-containing (+Ca^2+^) buffer (20 mM HEPES-Na, 150 mM NaCl, 0.005% Tween 20, 1 mM CaCl_2_, pH 7.9) at the third wash step. Beads were then rotated in the presence of 1 ml of 1.2-μM mouse CaM in −Ca^2+^ buffer or +Ca^2+^ buffer at 4°C for 2 h. After washing the beads with either the −Ca^2+^ buffer or +Ca^2+^ buffer, proteins on the beads were eluted by heating in an SDS-PAGE sample buffer. Protein samples were separated on 15% SDS-PAGE then transferred onto a 0.2-μm polyvinylidene fluoride (PVDF) membrane (GE Healthcare). Membranes were blocked with 2% BSA in TBS-T (20 mM Tris–HCl, 150 mM NaCl, 0.1% Tween 20, pH 7.5) at room temperature for 1 h, then incubated with an anti-calmodulin antibody (Millipore, cat no. 05–173, 1:1000) in TBS-T at 4°C overnight. This incubation was followed by incubation with peroxidase-labeled anti-mouse IgG (GE Healthcare) at room temperature for 1.5 h. Protein bands were developed using Immobilon Western Chemiluminescent HRP Substrate (Millipore).

For binding analysis designed to compare mCaM and ySUMO-fusion proteins of mT1rs CT, purified proteins dissolved in −Ca^2+^ buffer (20 mM Tris HCl, 150 mM NaCl, 0.1% Tween 20, pH 7.5) or +Ca^2+^ buffer (20 mM HCl 150 mM NaCl, 0.1% Tween 20, 1 mM CaCl_2_, pH 7.5) were prepared by dialysis. Ni-NTA Superflow (20 μl bed. vol.) was mixed with His-tagged ySUMO-fusion proteins in 500 μl of –Ca^2+^ buffer and rotated at 4°C for 2 h. The beads were then washed with Ca^2+^ buffer containing 6 M guanidine chloride to denature the ySUMO portion on the beads. The beads were then mixed with 0.6 μM of mCaM in 500 μl of –Ca^2+^ or +Ca^2+^ buffer and rotated at 4°C for 2 h. After washing the beads with –Ca^2+^ or +Ca^2+^ buffer, the proteins on the beads were eluted by heating in SDS-PAGE sample buffer. mCaM bands were detected by western blotting as described previously.

### Surface plasmon resonance analysis

Surface plasmon resonance (SPR) analysis was carried out using a Biacore 2000 biosensor (GE Healthcare). To measure the interaction between CaM and biotinylated T1r peptides, the peptides were immobilized on a CAP sensor chip using a Biotin CAPture Kit (GE Healthcare), with all procedures performed as per the manufacturer’s specifications. Sensorgrams were collected after infusing a 2-fold serial dilution series from 50 μM of CaM in −Ca^2+^ or +Ca^2+^ buffer at a flow rate of 30 μl/min and a constant temperature of 25°C. The surface was regenerated by an infusion of regeneration buffer from the Biotin CAPture Kit after each run. To measure the interaction of CaM with ySUMO-fusions of T1rs, the ySUMO-fusion proteins were immobilized on a CM5 sensor chip using an Amine Coupling Kit (GE Healthcare). ySUMO proteins were immobilized on the surface of a reference cell to prevent nonspecific interaction of the analytes with the surface. Sensorgrams were collected after infusing a 2-fold serial dilution series from 40 μM of CaM in −Ca^2+^ or +Ca^2+^ buffer at a flow rate of 30 μl/min and a constant temperature of 25°C. The surface was regenerated by infusion of 50 mM NaOH for 20 s at a flow rate of 100 μl/min. All obtained sensorgrams were processed and analysed using BIAevaluation version 1.3. Double-referenced sensorgrams were obtained by subtracting the response from the reference cell and subsequently subtracting the sensorgrams of the buffer (*i.e.* analyte concentrations of zero). Dissociation constants (*K*_D_) were estimated from plots of equilibrium response (*R*_eq_) values against mCaM concentrations by fitting a 1:1 binding model using Igor Pro software (WaveMetrics).

## Results

### CaM-binding motif in the T1r cytosolic C-term region

We first determined whether T1rs have CaM-binding motifs in their amino acid sequences. Because mGluRs have CaM-binding sites at the cytosolic C-terminal regions *(**24**)*, we predicted the boundaries between transmembrane regions and the cytosolic C-terminal regions in T1rs (Fig. 1A). We then subjected the amino acid sequences of the cytosolic regions of T1rs to CaM-binding site predictions. The TMHMM server defined cytosolic regions as residues 810–841 for hT1r1, 811–842 for mT1r1, 810–839 for hT1r2, 814–843 for mT1r2, 818–852 for hT1r3 and 823–858 for mT1r3. For T1r3, sequences upstream of predicted regions share a conserved motif with the predicted cytosolic regions in T1r1 and T1r2; this is referred to as P(K/R)CYΨΨΨ, where ‘Ψ’ is an amino acid with a large aliphatic side chain (Fig. 1B). Therefore, sequences starting from the conserved motif upstream of the initial prediction—*i.e.* 812–852 for hT1r3 and 817–858 for mT1r3—were used for further analyses.

As a result, a region around residues 822–842 in mT1r3 was identified as a presumable binding site because it received high scores from two different CaM-binding site prediction servers *(**31**,**32**)*. In contrast, regions in hT1r3, hT1r1 and mT1r1 received moderate scores from at least one of the prediction servers in the middle of this region (*i.e.* the Calmodulation database and Meta-analysis predictor) (Fig. 1B). These results suggested the presence of a CaM-binding site in the cytosolic C-terminal region of mouse T1r3 and possibly in other T1r subunits. Based on these predictions, the cytosolic C-terminal regions from mouse T1rs were subjected to further biochemical analyses.

### Direct binding of mCaM to mT1r cytosolic C-terminal regions

**Fig. 2:**
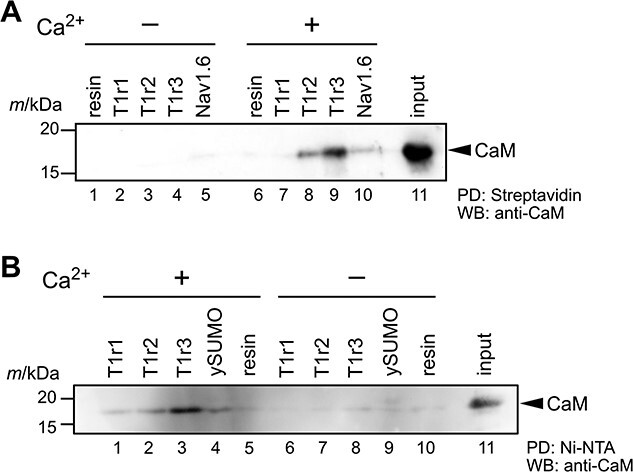
**Interaction between calmodulin and the cytosolic C-terminal regions of T1rs. (A)** Interaction of mouse calmodulin with T1r peptides. **(B)** Interaction of mouse calmodulin with ySUMO-fusion proteins of the T1r C-terminal region.

To examine the binding of calmodulin to the C-terminal region of T1rs, we first carried out a pull-down assay using chemically synthesized peptides of mT1r C-terminal sequences. These chemically synthesized peptides were designed to incorporate a biotin moiety at the N-terminal amino group for immobilization, a GGSGGS linker sequence and then the C-terminal region of T1rs (Fig. 1C). For the control sample, we also synthesized a Nav1.6 peptide comprising its CaM-binding region because Nav1.6 is known to interact with CaM in a Ca^2+^-independent manner *(**37**,**38**)*. Purified mouse CaM was then mixed with peptide-immobilized streptavidin-coated magnetic beads. The coprecipitated CaM protein was subsequently detected by western blotting (Fig. 2A). In the presence of Ca^2+^ ions, the noticeable bands corresponding to CaM were detected on mT1r2- and mT1r3-immobilized beads (Fig. 2A, lanes 8 and 9), whereas a slight band on the control beads was observed (Fig. 2A, lane 6). The level of CaM protein detected on mT1r1-immobilized beads was like that on the control beads (Fig. 2A, lane 7), suggesting that the affinity of mT1r1 to CaM is lower than the affinities of mT1r2 and mT1r3. On the other hand, bound CaM proteins were not detected for all mT1rs in the absence of Ca^2+^ ions. In contrast, an interaction between CaM and the Nav1.6 peptide was observed both in the presence and absence of Ca^2+^ ions (Fig. 2A, lanes 5 and 10). Taken together, these results suggest that CaM binds to mT1rs in a Ca^2+^-dependent manner.

**Fig. 3:**
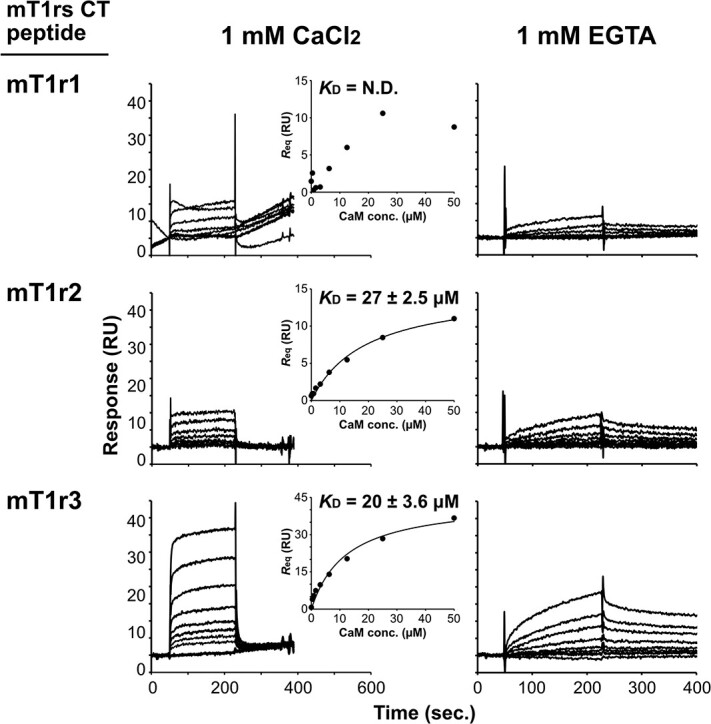
**SPR binding analysis of calmodulin to mT1r peptides.** Measurements were carried out in the presence (1 mM CaCl_2_) and absence (1 mM EGTA) of Ca^2+^. Shown are the sensorgrams of the interaction between calmodulin and mT1r CT peptides. In the panels of the 1 mM CaCl_2_ conditions, the insets show equilibrium responses plotted against the concentration of injected mCaM. The equilibrium dissociation constants (*K*_D_) were estimated by fitting the curve to a 1:1 binding model. The indicated errors indicate standard error determined from curve fitting.

We also tested the binding of CaM to the ySUMO-fusion proteins of the C-terminal region of T1rs by a pull-down assay. The purified ySUMO fusion proteins of mT1rs, termed FLAG-ySUMO-mT1rs CT (Fig. 1C, *middle*), were immobilized on the beads via their histidine tag. These immobilized proteins were then treated with guanidine chloride to avoid steric hindrance caused by the ySUMO portion before applying mCaM. Bound mCaM was observed for all T1r subtypes in the presence of Ca^2+^ ions (Fig. 2B, lanes 1–3), whereas the levels of detected mCaM were similar to that on the control beads for all T1rs in the absence of Ca^2+^ ions (Fig. 2B, lanes 6–8 vs lane 10). These results are consistent with those obtained from the pull-down assay and signifies that mCaM has the highest affinity for mT1r3 among the three subtypes.

### Surface plasmon resonance analysis of the interaction between CaM and T1rs

Having confirmed the interaction between mCaM and mT1rs CT, we next analysed these interactions using SPR analysis. The biotin-mT1rs CT peptides were immobilized on the sensor chip via streptavidin. The SPR signal was measured by injection of varying concentrations of mCaM (Fig. 3). SPR measurements in the presence of Ca^2+^ provided sensorgrams indicating the binding signals with concentration-dependent increase on the injected mCaM. They also showed the rapid association and dissociation of mCaM on mT1rs across all subtypes. Similar features in the sensorgrams have been reported in SPR measurements of the interactions between CaM and other CaM-binding motifs *(**39–42**)*. At the highest concentration of mCaM, the response at steady state (*R*_eq_) values for the interactions with mT1r1 and mT1r2 were both ~11 RU, whereas a value of 36.7 RU was observed for mT1r3. For reference, the immobilization levels of peptides on the sensor chip were 661, 81.7 and 86.8 RU for mT1r1, mT1r2 and mT1r3, respectively (Table 1). Based on these immobilization levels, the interaction between mT1r1 and mCaM seems to be the weakest among the three subtypes, whereas the affinity of mT1r3 with mCaM seems to be the strongest. In fact, the dissociation constant (*K*_D_) values were estimated using a steady-state binding model to be 27 and 20 μM for mT1r2 and mT1r3, respectively, whereas the *K*_D_ value for

**Table 1:** Immobilization levels and mCaM-binding responses of mT1rs CT peptides

	*R*_eq_ value or response at 50 μM mCaM injection (RU)	Immobilization level (RU)
**1 mM CaCl**_**2**_		
T1r1	10.6^*^	661
T1r2	11.0	81.7
T1r3	36.7	86.8
**1 mM EGTA**		
T1r1	5.0	137
T1r2	9.4	68.6
T1r3	16.0	96.5

^*^ *R*_eq_ value was obtained with 25 μM mCaM injection.

mT1r1 was a failure in estimation due to the low signals (Fig. 3). However, it should be noted that the observed *K*_D_ values are relatively high, indicating that the affinities for CaM-T1rs interactions are weak even for mT1r2 and mT1r3.

**Fig. 4:**
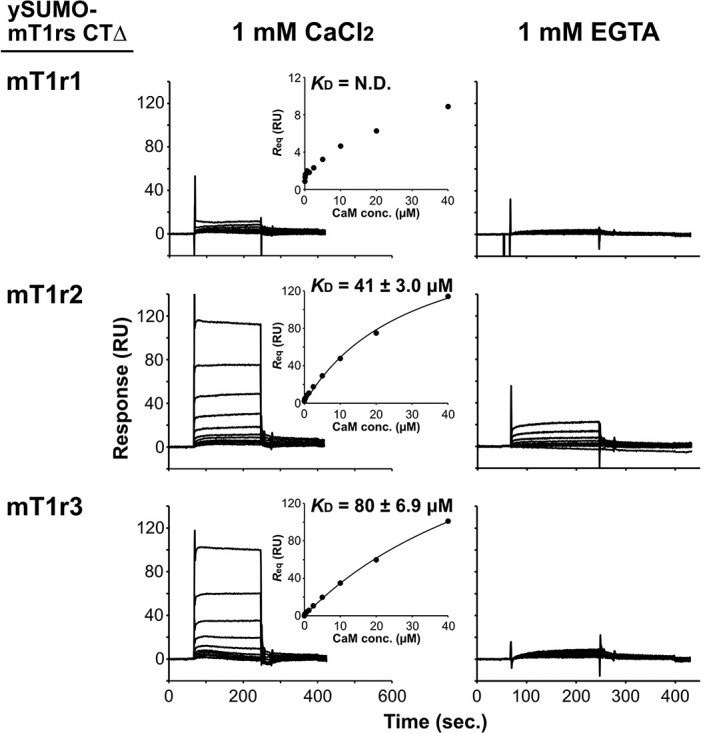
**SPR binding analysis of calmodulin to ySUMO fusion proteins of the mT1r C-terminal region.** Measurements were carried out in the presence of Ca^2+^ (1 mM CaCl_2_) and in the absence of Ca^2+^ (1 mM EGTA). Shown are sensorgrams for the interaction of calmodulin with ySUMO-mT1rs CTΔ proteins. The equilibrium responses plotted against the concentration of injected mCaM and estimated *K*_D_ values and standard errors are shown in the insets as in Fig. 3.

To investigate the effect of Ca^2+^ on these interactions, we next performed SPR measurements in the absence of Ca^2+^ (*i.e.* in a buffer containing 1 mM EGTA). These measurements yielded sensorgrams that show slow binding and dissociation for all mT1r subtypes, with binding responses increasing according to the concentration of injected mCaM. The profiles of the interactions between CaM and T1rs were found to be different in the presence and absence of Ca^2+^; the latter showed slower association and dissociation kinetics compared with the former. At the highest mCaM concentration, the responses for mT1r1, mT1r2 and mT1r3 were ~5.0, ~9.4 and ~16 RU, respectively. The immobilization levels of T1rs peptides on the sensor chip were 137 RU for mT1r1, 68.6 RU for mT1r2 and 96.5 RU for mT1r3, respectively. Given these immobilization levels of T1r peptides, we conclude that the maximum number of CaM-binding sites for T1rs peptides was smaller in the absence of Ca^2+^ than in its presence. Moreover, this was especially for T1r3. All these results confirmed the results of the pull-down assay.

Next, we carried out further SPR measurements using a ySUMO-fusion form of the C-terminal region of mT1rs. Because the synthesized peptides were observed to be aggregated on the sensor chip, we first designed and prepared ySUMO-mT1rs CTΔ, in which the short segment containing the PKCY(V/M) conserved residues in the N-terminal portion was omitted to improve solubility (Fig. 1C, *bottom*). As a result, we were able to observe obvious binding signals of mCaM to immobilized ySUMO-mT1r2 CTΔ and mT1r3 CTΔ, whereas we detected only a slight binding signal to ySUMO-mT1r1 CTΔ in the presence of 1 mM Ca^2+^ (Fig. 4, *left*). The estimated *K*_D_ values of CaM binding to T1r2 and T1r3 were 41 and 80 μM, respectively. These are comparable with the *K*_D_ values estimated from measurements using synthetic peptides. Taken together, these results suggest that CaM mainly recognizes regions other than the PKCY(V/M) sequence in T1rs CT. Furthermore, the CaM-binding signals were lower in the absence of Ca^2+^ than in its presence (Fig. 4, *right*). ySUMO-mT1r1 CTΔ showed a weak response—*i.e.* ~4.4 RU at the highest concentration of mCaM. ySUMO-mT1r2 CTΔ and ySUMO-mT1r3 CTΔ both showed more significant responses than ySUMO-mT1r1 CTΔ but smaller responses than those observed in the presence of Ca^2+^ (Table 2). These results also suggest that the interaction between mCaM and ySUMO-mT1r CTs depends on the presence of Ca^2+^.

**Table 2:** Immobilization levels and mCaM-binding responses of ySUMO-mT1rs CT proteins

	*R*_eq_ value or response at 40 μM mCaM injection (RU)	Immobilization level (RU)
**1 mM CaCl**_**2**_		
T1r1	11.1	1568
T1r2	114	1811
T1r	101	1468
**1 mM EGTA**		
T1r1	4.4	1568
T1r2	9.2	1811
T1r3	13	1468

## Discussion

In this study, we found evidence of direct binding of CaM to the C-terminal cytosolic regions of T1rs—and specifically to those of mouse T1r3 and T1r2—in a calcium-dependent manner. Obvious binding signals of CaM to T1rs were also observed but these were significantly weaker in the absence of Ca^2+^ compared with its presence (Figs. 3 and Fig. 4, Tables 1 and 2), indicating that the presence of Ca^2+^ may enhance the interaction between CaM and T1rs. On the other hand, binding and dissociation rates in the absence of Ca^2+^ were slower than in the presence of Ca^2+^. For reference, we also attempted to estimate the values of kinetic parameters, such as the association rate constant (*k*_a_) and the dissociation rate constant (*k*_d_) by a global fitting to a 1:1 binding model. The *K*_D_ values for the Ca^2+^-free samples calculated from *k*_a_ and *k*_d_ were moderately smaller than those determined in the presence of Ca^2+^, although it should be noted that the curve fits were poor, and the estimation method was different from that used for the samples with Ca^2+^ (Supplementary Material, Fig. S1). The results obtained in this study were limited by the fact that they were determined by the C-terminal regions of T1rs in peptide or fusion-protein formats. Currently, difficulty in protein sample preparation of full-length T1rs hampers accurate analyses of the affinity of CaM to T1rs under physiological conditions. Nevertheless, given that the cellular CaM concentration ranges several to ~25 μM *(**43**)* and assuming that *in vivo* CaM affinities to T1rs are comparable with those determined in this study, our results suggest that a small to considerable population of T1rs could represent a CaM-bound state. Moreover, considering the differences in binding signals and kinetics observed in the presence and absence of Ca^2+^, our observations suggest that CaM binds T1rs in different manners—*i.e.* in terms of occupancy and/or duration—according to cytosolic Ca^2+^ concentration. The effect of the CaM binding would depend on the signaling kinetics for the receptor and downstream molecules and/or local concentration of the participating molecules and thus should be experimentally examined.

Although the full-length structure of T1rs has not yet been elucidated, CaM-binding sites have been deduced to be located just beneath the plasma membrane because the C-terminal cytosolic tails of T1rs are relatively short, with only ~30–40 amino acid residues (Fig. 1B). Recent structural analyses of other class C GPCRs have revealed their full-length structures and thereby provide a possible mechanism for signal transduction by this type of receptor *(**10**,**44**,**45**)*. For members of this class, an agonist binding to the LBD first induces a conformational change accompanying dimer rearrangement. This is followed by reorientation of the transmembrane domains, resulting in G protein activation on the cytosolic side. Assuming that T1rs use this activation mechanism along with other class C GPCRs, CaM binding to their C-terminal cytosolic tails might sterically affect the receptor activation process accompanying with reorientation of the transmembrane domains and/or G-protein binding and activation process on the cytosolic face of the receptor. This presumption applies to both sweet (T1r2/T1r3) and umami (T1r1/T1r3) receptors because T1r3 exhibited the highest binding activity to CaM and is the common subunit for both. Because signaling cascades downstream of T1rs result in cytosolic Ca^2+^ release from the endoplasmic reticulum *(**46**)*, we speculate that Ca^2+^-dependent binding of CaM to T1rs observed in this study might serve as a feedback regulation system for T1r signaling, although its direction may be positive or negative. In addition, the possibility of regulation of processes other than signaling, such as membrane trafficking, as has been observed for mGluRs *(**28**,**29**)*, may also be present. In summary, the finding of CaM binding to T1rs implies a possibility of the existence of a T1r regulatory system on the cytosolic side, and this has not yet been extensively characterized. Since no physiological evidence has so far been reported, future studies verifying the hypothesis are required.

The observations in this study also suggest that regulation of T1rs by CaM, if any, may have diverse effects. CaM shows distinct binding activities to different subtypes of T1rs: here, T1r3 showed the highest binding activity, T1r2 showed a slightly lower but comparable activity to T1r3, whereas T1r1 showed a low, near-insignificant binding signal. These results imply that the regulatory effects of CaM on different heterodimeric receptors—*i.e.* the sweet (T1r2/T1r3) and umami (T1r1/T1r3) receptors—may be different. Even if we focus on T1r3, the predicted scores for CaM binding were found to differ between mouse and human ones (Fig. 1B). An extended CaM-binding site prediction on T1rs of other mammalian and representative vertebrate species showed that the C-termini of T1r3s tend to receive the highest prediction scores in many species, especially in rodents, whereas most of the other T1rs also receive moderate scores at the corresponding regions exhibiting positive scores in mouse and human T1rs (Supplementary Material, Data S1). The result suggests that the CaM-binding property might be a common property of T1rs, especially of T1r3s. Nevertheless, because the scores values vary among species, the actual CaM binding on T1rs in species other than mouse should be examined in future studies. Furthermore, T1rs are expressed not only in taste buds but also in various cells and tissues throughout the body *(**47**,**48**)*. Because expression levels of CaM and Ca^2+^ dynamics likely vary among tissues and cells, CaM effects on non-taste T1rs present in these locations may also vary. This study focused on the intermolecular interactions between limited regions of T1rs and CaM *in vitro*, and the physiological relevance of CaM binding to T1rs in terms of receptor function should be examined by future studies.

## Supplementary Material

Web_Material_mvad060
