# Supplementary material for: Direct binding of calmodulin to the cytosolic C-terminal regions of sweet/umami taste receptors
Source: J Biochem. 2023 Aug 1;174(5):451–9. doi: 10.1093/jb/mvad060 (PMC11033526; doi:10.1093/jb/mvad060)
Supplement: Web_Material_mvad060 [file web_material_mvad060.pdf]

Supplementary Information for:

Direct binding of calmodulin to the cytosolic C-terminal regions of sweet/umami taste receptors

Atsuki Yoshida, Ayumi Ito, Norihisa Yasui, Atsuko Yamashita

Graduate School of Medicine, Dentistry and Pharmaceutical Sciences, Okayama University, 1-1-1, Tsushima-naka, Kita-ku, Okayama, 700-8530, Japan

Corresponding Authors

Norihisa Yasui

E-mail: nyasui@okayama-u.ac.jp

Atsuko Yamashita

E-mail: a_yama@okayama-u.ac.jp

1 mM EGTA

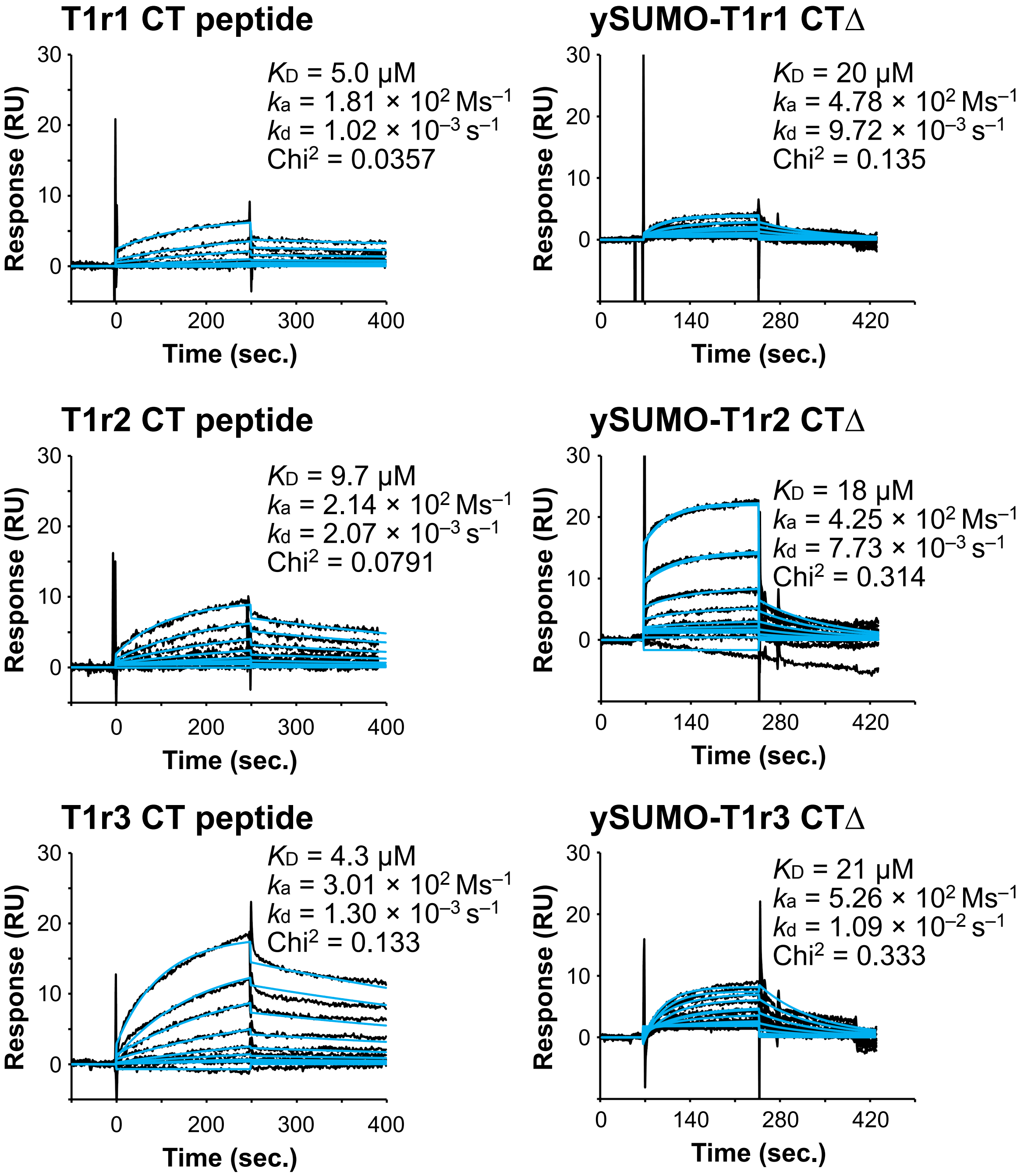

Figure S1. Surface plasmon resonance kinetic analysis of the CaM-T1rs interaction in the absence of Ca^{2+}. Shown are sensorgrams obtained from measurements in the absence of Ca^{2+} (in a buffer containing 1 mM EGTA). These are identical to the ones presented in Figures 3 and 4. The kinetic parameters, the association rate constant (k_a), and dissociation rate constant (k_d) were estimated by global fitting to a 1:1 binding model using BIAevaluation version 1.3. Actual binding curves (black) are overlaid with the fitted curves (cyan). Dissociation constant (K_D) values were calculated using the k_a and k_d values estimated by curve fitting.

Data S1. Amino acid sequences of the C-terminal regions of T1rs and the scores of the Calmodulin Target Database (Pr1) and the Calmodulation database and Meta-analysis predictor (Pr2) servers.

Human (*Homo sapiens*; Mammalia / Placentalia / Euarchontoglires / Euarchonta / Primate)

```
T1r1- PKCYVILCRP DLNSTEHFQA SIQDYTRRCG ST
(Pr1) 0000000000 0000000000 0000000000 00
(Pr2) 0000224444 4444444411 1100000000 00
T1r2- PKCYMILFYP ERNTPAYFNS MIQGYTMRRD
(Pr1) 0000000000 0000000000 0000000000
(Pr2) 0000002222 2222222211 1100000000
T1r3- PRCYLLMRQP GLNTPEFFLG GGPGDAQGQN DGNTGNQGKH E
(Pr1) 0000000000 0000000000 0000000000 0000000000 0
(Pr2) 0000244444 4444444310 0000000000 0000000000 0
```

Mouse (*Mus musculus*; Mammalia / Placentalia / Euarchontoglires / Glires / Rodentia)

```
T1r1- PKCYVILCRP ELNNTEHFQA SIQDYTRRCG TT
(Pr1) 0000000000 0000000000 0000000000 00
(Pr2) 0000224444 4444444411 1100000000 00
T1r2- PKCYMILFYP ERNTSAYFNS MIQGYTMRKS
(Pr1) 0000000000 0000000000 0000000000
(Pr2) 0000002222 2222222211 1100000000
T1r3- PKCYVLLWLP KLNTQEFFLG RNAKKAADEN SGGGEAAQGH NE
(Pr1) 0000003699 9999999999 9999996300 0000000000 00
(Pr2) 00003569AA AAAAAAA720 0000000000 0000000000 00
```

Rat (*Rattus norvegicus*; Mammalia / Placentalia / Euarchontoglires / Glires / Rodentia)

```
T1r1- PKCYVILCRP ELNNTEHFQA SIQDYTRRCG TT
(Pr1) 0000000000 0000000000 0000000000 00
(Pr2) 0000224444 4444444411 1100000000 00
T1r2- PKCYMILFYP ERNTSAYFNS MIQGYTMRKS
(Pr1) 0000000000 0000000000 0000000000
(Pr2) 0000002222 2222222211 1100000000
T1r3- PKCYVLLWLP ELNTQEFFLG RSPKEASDGN SGSSEATRGH SE
(Pr1) 0000000000 0000000000 0000000000 0000000000 00
(Pr2) 00003569AA AAAAAAA720 0000000000 0000000000 00
```

Rabbit (*Oryctolagus cuniculus*; Mammalia / Placentalia / Euarchontoglires / Glires / Lagomorpha)

```
T1r1- PKCYVILCRP DLNSTEHFQA SIQDYTRRCG SS
(Pr1) 0000000000 0000000000 0000000000 00
(Pr2) 0000224444 4444444411 1100000000 00
T1r2- PKCYLILFFP ERNTPSYFNS VIQGYTMRRD
(Pr1) 0000000000 0000000000 0000000000
(Pr2) 0000124577 7777777744 4200000000
T1r3- PKCYLLLQQP QLNTAEFFLG ADPRDVRGGG GGGEEVQGSH G
(Pr1) 0000000000 0000000000 0000000000 0000000000 0
(Pr2) 0000245555 5555557632 2222220000 0000000000 0
```

Bovine (*Bos taurus*; Mammalia / Placentalia / Laurasiatheria / Cetartiodactyla)

```
T1r1- PKCYVILYRP DLNSTEHFQA SIQDYTRRCG ST
(Pr1) 0000000000 0000000000 0000000000 00
(Pr2) 0000224444 4444444411 1100000000 00
T1r2- PKCYMILFYP ERNTPAYFNS VIQGYTMRKD
(Pr1) 0000000000 0000000000 0000000000
(Pr2) 0000013444 4444444433 3100000000
T1r3- PKCYLLLWRP DLNTPEFFLG GGPSDARGQG GSGHGEETQG KNK
(Pr1) 0000000000 0000000000 0000000000 0000000000 000
(Pr2) 0000245888 8888888520 0000000000 0000000000 000
```

Dog (*Canis lupus familiaris*; Mammalia / Placentalia / Laurasiatheria / Carnivora)

```
T1r1- PKCYVILCRP DLNSTEHFQA SIQDYTRRCG ST
(Pr1) 0000000000 0000000000 0000000000 00
(Pr2) 0000224444 4444444411 1100000000 00
T1r2- PKCYMVLFYP ERNTQVYFSS MIQGYTMGKD
(Pr1) 0000000000 0000000000 0000000000
(Pr2) 0000003333 3333332211 1100000000
T1r3- PKCYLLLQQL ELNNPEFFLG DDARGQGSSG SGGKETGKNK
(Pr1) 0000000000 0000000000 0000000000 0000000000
(Pr2) 0000245556 6666666520 0000000000 0000000000
```

Opossum (*Monodelphis domestica*; Mammalia / Marsupialia / Didelphimorphia)

```
T1r1- PKCYVILCRP DLNTTEYFQA SIQDYTQRCS SP
(Pr1) 0000000000 0000000000 0000000000 00
(Pr2) 0000224444 4444444411 1100000000 00
T1r2- PKCYMILFYP QRNTLAYFNN VIQGYTMRKE
(Pr1) 0000000000 0000000000 0000000000
(Pr2) 0000024666 6666655544 4100000000
T1r3- PKCYLLLWEP HLNTQEFFQS SLELGPRASG EAQEEKPEKR RSQ
(Pr1) 0000000000 0000000000 0000000000 0000000000 000
(Pr2) 0000235777 7777777411 1100000000 0000000000 000
```

Chicken (*Gallus gallus*; Aves)

```
T1r1- PKGFVVLLRP HLNTAERFQQ EIRSYTRRRD E
(Pr1) 0000000000 0000000000 0000000000 0
(Pr2) 0000224444 4444444522 2211111111 1
T1r3- PKCYILLLKP DLNREDYFQY STKEEPEGDP Q
(Pr1) 0000000000 0000000000 0000000000 0
(Pr2) 0000223333 3333333411 1111111111 1
```

Turtle (*Pelodiscus sinensis*; Reptilia)

```
T1r1- PKGYIILFRT ELNTTEHFQL SIQSYTQKKN SD
(Pr1) 0000000000 0000000000 0000000000 00
(Pr2) 0000336666 6666666633 1100000000 00
T1r2- PKCYVIFFHP ERNTAAYFQT AIQSYTMRQN
(Pr1) 0000000000 0000000000 0000000000
(Pr2) 0000113333 3333333311 1100000000
T1r3- PKCYILLFKP EWNTVAYFQS YAKEEPQEKD SR
(Pr1) 0000000000 0000000000 0000000000 00
(Pr2) 0000234444 4444433411 1111111111 10
```

Caecilian (*Rhinatrema bivittatum*; Amphibia)

```
T1r1- PKCYVILLRK DLNTPEHFQT SIQNYTRKKN SD
(Pr1) 0000000000 0000000000 0000000000 00
(Pr2) 0000224444 4444444411 1100000000 00
T1r3- PLCYIILFKP ELNTLSYFPS TAEEPPGEQQ DSRSTQLQN
(Pr1) 0000000000 0000000000 0000000000 000000000
(Pr2) 0111345555 5555533300 0000000000 000000000
```

Medaka (*Oryzias latipes*; Osteichthyes)

```
T1r1- PKIYIIVLKP KMNTTAHFQN CIQMYTMNK
(Pr1) 0000000000 0000000000 000000000
(Pr2) 0011224444 4444444411 110000000
T1r2a PKCYIIIFKP DKNTQQYFQG LIQSYTKTIS Q
(Pr1) 0000000000 0000000000 0000000000 0
(Pr2) 0000124555 5555555644 4211111110 0
T1r3- PKCFLLLRKP NLNTPQQFCT FLEGVPPTQT EEEPQPRQEK
(Pr1) 0000000000 0000000000 0000000000 0000000000
(Pr2) 0000235555 5777777744 4211100000 0000000000
```

Database IDs for amino acid sequences

Species	T1r1	T1r2	T1r3
Homo sapiens	Q7RTX1	Q8TE23	Q7RTX0
Mus musculus	Q99PG6	Q925I4	Q925D8
Rattus norvegicus	Q9Z0R8	Q9Z0R7	Q923K1
Oryctolagus cuniculus	G1TTE4	G1SZQ9	XP_051696443.1*
Bos taurus	E1BPM8	F1N5V2	A0A3Q1NND8
Canis lupus familiaris	A0A8C0MNP5	Q49HI0	A0A8I3PRQ9
Monodelphis domestica	XP_001377313.1*	XP_056681894.1*	XP_007481142.2*
Gallus gallus	A0A088DBQ4	–	XP_040544961.1*
Pelodiscus sinensis	XP_006123180.1*	XP_006115160.1*	XP_006127185.1*
Rhinatrema bivittatum	XP_029433915.1*	N/A	XP_029433926.1*
Oryzias latipes	BAE78481.2*	BAE78482.2*†	BAE78485.1*

Sequences derived from Uniprot database were used, except that those marked with * were obtained from Genbank.

-: no gene. N/A: The full-length sequence is not available. †: T1r2a.
